# Management of Benign Paroxysmal Positional Vertigo with the TRV Chair

**DOI:** 10.1055/a-2795-9986

**Published:** 2026-09-11

**Authors:** Sandra Krzywdzińska, Michał Kaczmarczyk, Paweł Rozbicki, Dariusz Jurkiewicz, Jacek Usowski, Marcin Jadczak

**Affiliations:** 1 Department of Otolaryngology with Division of Cranio-Maxillo-Facial Surgery, Military Institute of Medicine-National Research Institute, Warsaw, Poland

**Keywords:** vertigo, repositioning maneuvers, BPPV, TRV chair, VNG

## Abstract

**Introduction:**

Despite the growing awareness regarding benign paroxysmal positional vertigo (BPPV), establishuing an accurate diagnosis in atypical or subtle cases remains a clinical challenge, especially when standard positional maneuvers yield negative results.

**Objective:**

To evaluate the effectiveness of the TRV Chair (Interacoustics A/S) in the diagnosis and treatment of patients with a clinical history suggestive of BPPV but negative findings on conventional diagnostic maneuvers.

**Methods:**

A total of 40 patients with vertigo symptoms suggestive of BPPV and negative results on standard positional tests underwent evaluation and therapy using the TRV Chair. Symptom severity and functional impact were assessed before and after the intervention using the Dizziness Handicap Inventory (DHI; total and subscales), the Vertigo Symptom Scale (VSS), and the Berg Balance Scale (BBS).

**Results:**

Significant improvements were observed following the TRV Chair intervention. The mean total DHI score decreased from 40.3 to 22.2 ( *p* < 0.0001), with improvements in the functional (17.6–7.1), emotional (13.5–8.8), and physical (17.1–5.8) subscales ( *p* < 0.0001 for all). The VSS scores improved from 17.7 to 10.4 ( *p* < 0.0001), and the BBS scores increased from 51.8 to 53.6 ( *p* < 0.0001).

**Conclusion:**

The TRV Chair appears to be an effective diagnostic and therapeutic tool for patients with suspected BPPV not confirmed by standard tests. Its application may enhance the management of atypical cases; however, further studies are warranted to define optimal clinical protocols and confirm the long-term efficacy.

## Introduction

Benign paroxysmal positional vertigo (BPPV) is currently one of the most common reasons behind peripheral vertigo. At present, it is estimated that BPPV constitutes 14 to 42% of all instances of vertigo. 1 2 3 The variability in the occurrence of this phenomenon, depending on the medical center, can be explained by misdiagnosis or missed diagnosis, which is why the statistic seems understated. It is estimated that the frequency of vertigo occurrence in a lifetime is of 2.4%, and it is 2 times more frequent in female than in male individuals. Studies 1 have reported that the highest incidence rate falls between the ages of 50 and 70 years; nonetheless, BPPV occurs in all age groups.

The complete picture of BPPV consists of a sudden, temporary sense of either vertical or horizontal rotational vertigo evoked by a particular head positioning or changing of body position. It is important to note that, contrary to numerous instances of central vertigo, BPPV is not a life-threatening condition, with symptoms resolving spontaneously within a few weeks and with a tendency to reoccur, which has a significant impact on the quality of life of the patient and their environment. 4 In some cases, recurrent episodes may lead to anxiety, avoidance of movement, and increased risk of falls, especially among elderly individuals. Approximately 90% of BPPV instances are idiopathic; the secondary causes of this phenomenon are head injuries (17%), vestibular neuronitis (15%), vertebrobasilar insufficiency, labyrinthitis, and complications of middle-ear surgery. 5

At present, BPPV can be classified depending on the semicircular canal affected, with a division into posterior-canal BPPV (p-BPPV), lateral-/horizontal-canal BPPV (h-BPPV), anterior-canal BPPV (a-BPPV), and multicanal and bilateral multicanal BPPV, or depending on the pathology formation mechanism, with a division into canalolithiasis (CAN) and cupulolithiasis (CUP). 2 3 6 Correct canal and mechanism identification is essential to select the most effective repositioning maneuver.

A typical diagnosis is based on anamnesis obtained from a patient and a positive result of diagnostic maneuvers performed—the Dix–Hallpike (DHP) maneuver or the Supine Roll test, depending on the canal affected. 2 4 Unfortunately, diagnosis and subsequent treatment are often problematic. It appears that, due to existing limitations such as a high body weight of the patient, cervical spine disorders, or simply lack of cooperation between the patient and the doctor, in some patients, diagnostic maneuvers cannot be performed. Problems may also occur when attempting to diagnose more complicated instances, such as multicanal BPPV. It is suspected that typical methods are insufficient for the diagnosis of up to 10 to 20% of the cases, although several authors 5 7 report an even greater proportion. Furthermore, the accuracy of bedside maneuvers is highly dependent on the clinician's experience and the patient's physical capabilities, both of which may influence test reliability.

In terms of treatment, traditionally, canalith repositioning procedures (CRPs) remain the first-line approach. The Epley maneuver is used most frequently for p-BPPV, while the Lempert (barbecue [BBQ] roll) maneuver is applied in h-BPPV cases. Less common variants, such as a-BPPV or CUP, often require modified or repeated procedures. In many cases, a single maneuver suffices, but in recurrent or resistant BPPV, repeated or alternative strategies may be necessary.

**Fig. 1:**
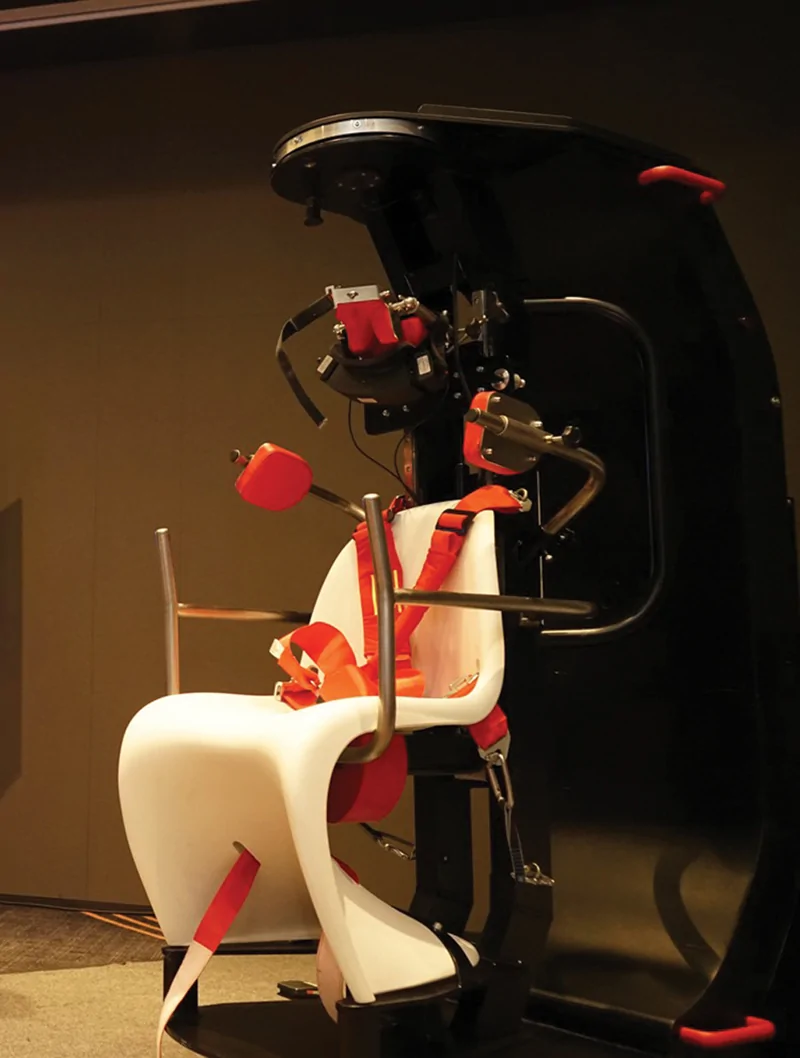
Illustration of the TRV Chair.

To meet the challenges of BPPV diagnosis, Thomas Richard-Vitton, MD, invented the TRV Chair 13 (Interacoustics A/S; Fig. 1 ), which enables the rotation of the patient along the plane of all semicircular canals, while simultaneously using infrared videonystagmography (VNG) goggles to monitor eye movement. 8 The action mechanism of this device eliminates the rotation of the neck and back in patients during the maneuvers, and it also nullifies the lack of cooperation element or subconscious resistance from the patient during the doctor's attempts to turn their head. 7 9 10 The purpose of the TRV Chair is to facilitate the diagnosis, detect even slight nystagmus, and consequently perform treatment maneuvers even in problematic patients who cannot have traditional maneuvers performed on them. It also enables real-time observation of eye movements with high precision, improving diagnostic sensitivity and canal location. Additionally, the chair supports immediate therapeutic intervention following diagnosis, increasing treatment efficiency in a single session. Apart from the usual maneueversm the TRV chair enables dynamic particle pepositioning maneuvers (DPRM) of the BBQ roll and the Semont and Epley maneuvers. The goal of those are causing sudden accelerations and decelerations of the patients and, therefore, of their otoliths. The mechanism assumes that after a forced stop, the otoliths are more rapidly reintroduced into their typical position, thus the maneuvers are more effective.

In the clinical practice, the TRV Chair proves especially valuable for elderly patients, individuals with orthopedic or neurological limitations, and those with atypical or multicanal involvement.

The current study aimed to assess the usefulness of the TRV Chair in the diagnosis of patients suffering from vertigo—with indications of BBPV–while demonstrating the ineffectuality of conventional diagnosing and, subsequently, to perform relevant treatment maneuvers with the use of the TRV Chair.

## Methods

Prospective studies were conducted with the use of the TRV Chair on 87 (61 female and 26 male) patients age between 18 and 80 years admitted to the Audiology Clinic of the Department of Otolaryngology and Laryngological Oncology with the Clinical Department of Craniofacial Surgery, Military Institute of Medicine, National Research Institute, in Warsaw, Poland, due to the occurrence of vertigo, with medical reconnaissance implying BPPV (vertigo connected with position, defined as a spinning sensation evoked by changes of position against the force of gravity, typically short-lasting [seconds], and occurring when a patient rotates their head/looks up or rolls to their side), and with a negative result of traditionally-performed diagnostic maneuvers. 8 11 12 All patients were referred from other hospitals or otolaryngology centers because of failure in prior diagnosis.

The inclusion criteria were: anamnesis referring to BPPV, previously-performed manual maneuvers, and positive diagnostic maneuvers performed with the TRV Chair 13 (DHP or Supine Roll test, depending on the affected canal). The division of BPPV into subtypes followed the premises of the current literature. 6 Moreover, anterior canalithiasis in patients with apogeotropic nystagmus (DHP) was distinguished in the current study.

The exclusion criteria were: height above 195 cm and below 140 cm, weight above 150 kg, age under 18 and over 80 years, history of cardio-/neurosurgical procedure within the previous month, OUN neoplastic diseases, headaches, positive skew test, vertigo unrelated to BPPV correctly diagnosed during the first visit, and a positive result of manual diagnostic maneuvers for BPPV during the first visit to our center.

**Fig. 2:**
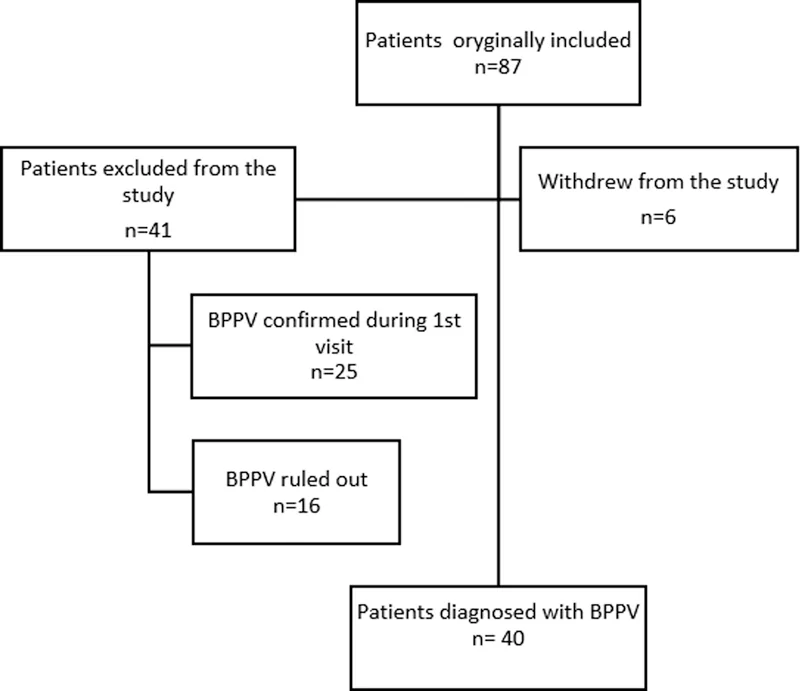
Flow diagram of the inclusion and exclusion of patients.

Patients received feedback verbally and in writing. Conscious agreement was obtained from all study participants. The research was approved by The Ethical Committee of the Military Chamber of Physicians, under Act 223/22. Inclusion of patients lasted from the 15th of May, 2022 until the 30th of June, 2023. After BPPV was confirmed, the subtype was identified, and further treatment in the TRV Chair was chosen. Out of 87 patients, 41 were excluded from the study; in 25 individuals, BPPV was diagnosed during the first visit with the help of traditionally-performed diagnostic maneuvers, and, in 16 patients, it was ruled out in the TRV Chair, and they were subjected to further examination. A total of 6 patients did not attend an appointment or decided to withdraw from the study ( Fig. 2 ).

Out of 40 patients included in the study, there were 31 female (77.5%) and 9 male (22.5%) subjects. The mean age of the patients during the first treatment was 58.4 ± 16.5. After determining the location of the affected canal, therapeutic maneuvers dedicated to decreasing stimulation for a given semicircular canal were performed. The maneuvers are predetermined and consistent with diagnosis and treatment for the use of the TRV Chair. At the beginning of each session and 3 weeks after each examination, the patients filled in the questionnaires–the Dizziness Handicap Inventory (DHI) and the Vertigo Symptom Scale (VSS). The Berg Balance Scale (BBS) was also used. After conducting the study, the enrolled patients remained under the care of our department. The improvement in the follow-up examination (patient's subjective experience, interview, and the aforementioned questionnaires), and the lack of abnormalities (positional vertigo) observed during the maneuvers, with lack of objective results (nystagmus), were considered good treatment outcomes of the treatment.

The TRV Chair 13 used in the current study is a mechanical, diagnostic, and repositioning tool created for the management of BPPV. It enables health professionals to rotate the patients 360° along the plane of each semicircular canal. It also gives the opportunity to lock the patient in any position for a detailed look at each semicircular canal, which enables the precise stimulation, diagnosis, and further treatment of BPPV subtypes. The effectiveness of the maneuvers can be increased by following the exact plane of the canal, and by adding kinetic energy to the liberatory maneuvers (kinetic energy also plays a very important role in the BBQ roll for patients with h-BPPV, making the treatment more effective). The chair comes with integrated VNG goggles, which eliminate visual fixation and enable magnification, analysis, and storage of nystagmus patterns during positioning. The availability of VNG goggles and the possibility of holding the patient stably reduces concerns about the incorrect performance of the maneuvers and interpretation, which would entail an incorrect application of these maneuvers. The VNG goggles minimize interference related to movements, enhancing the precision of the outcome of the maneuvers performed.

The diagnoses of p-BPPV, a-BPPV, and h-BPPV are performed with the DHP maneuver and the Supine Roll test. The criterion for BPPV is a position-dependent positional nystagmus with or without fatigue. Treatment with the TRV Chair is based on the Epley Maneuver for p-BPPV, the BBQ roll for h-BPPV (180–270 rotations), and the Epley Maneuver for the affected contralateral ear (reverse Epley) or other less-used or unique new maneuvers (such as dynamic maneuvers, the forward flip, the bend-over, and the Lorin Maneuver) for a-BPPV. 3 14 15 In the case of multicanal BPPV, the management first involves treating canalolithiasis, then, cupulolithiasis, and repositioning of the otoliths of the posterior canal before the horizontal canal. 3 13 The TRV Chair has an additional potentiated “impact” function which further enhances the effect of the maneuvers. 3

The DHI is a disability questionnaire frequently used in cases of vertigo. This survey, consisting of 25 items, was compiled due to the absence of a tool enabling the specific identification of the patients' functional, mental, and emotional problems related to dizziness. It helps to determine difficulties in the everyday functioning of the patients. The questionnaire evaluates functional (DHI-F – 36 points), physical (DHI-P – 28 points), and emotional (DHI-E – 36 points) disorders. For each question, the respondent can score 0 (no), 2 (sometimes), or 4 (yes) points and acquire a total number of points from 0 (no vertigo) to 100 (maximum handicap). The score enables the following classification of handicap: 16–34 points – mild handicap; 35–52 points – moderate handicap; ≥ 53 points – severe handicap. 16 17

The VSS is a questionnaire evaluating symptoms of imbalance, anxiety disorders, and autonomic dysfunctions in patients with vertigo. There are two variations of the scale: the long version (VSS-lv) and the short form (VSS-sf). The VSS-lv scale consists of 34 items developed based on medical interviews with patients. It evaluates the prevalence rate of the symptoms in the previous year, which is useful in epidemiological research. The VSS-sf comprises 15 items, and it was developed to measure the frequency of symptoms over the preceding month, and its main purpose is the evaluation of the therapeutical effects. 18 Kondo et al. 18 used the VSS-sf, covering a period of 1 month before the examination. The scale evaluates severe vertigo, short-term isolated dizziness, imbalance, and autonomic symptoms such as nausea, vomiting, headache, palpitation, and psychosomatic symptoms. Patients score points depending on the frequency of the symptoms, in the range from 0 points (symptoms never occur) to 4 points (symptoms occur more than once a week). Based on the intensification of autonomic and psychosomatic disorders, the scale enables the prognosis of the handicap degree within the next few months. 19

The BBS is a high-validity and high-reliability tool used to assess functional balance. Originally used in elderly patients, it proved to be a useful tool in assessing balance also in patients with amputations, neurological disorders (including traumatic brain injury), and acquired diseases such as strokes or multiple sclerosis. This scale also proved useful in assessing fall risks and anticipating the duration of in-hospital rehabilitation. 20 21 The BBS is a short test consisting of 14 mobility tasks of varied difficulty, which may be performed in a relatively-short time in different environments without high-tech equipment. 22 Data obtained from the surveys was statistically analyzed, and the results were compared.

### Statistical Analysis

The statistical analysis was conducted using the software STATISTICA software, version 7.0 Polish Language (PL; StatSoft. Inc). Visual assessment and the Kolmogorov–Smirnov test were used to verify the normal distribution of variables. In the Kolmogorov–Smirnov test, *p* > 0.05 was deemed the cut-off value. Normal distribution was confirmed for the DHI-P, DHI-F, and DHI-E variables. No simultaneous normal distribution was confirmed for the remaining variables before and after the intervention. The Student's *t* -test was used for variables with a normal distribution, while, for the remaining variables, the Mann–Whitney U-test was employed. The significance threshold was *p* < 0.05. The significance of the differences was confirmed for all variables examined.

## Results

Out of 87 patients examined with the use of the TRV Chair, BPPV was diagnosed in 40 (45.9%) individuals, including 31 femals and 9 male subjects with a mean age of 58.43 ± 16.54. The patients were then subjected to subsequent treatment maneuvers. In total, 41 patients were excluded from the study during the diagnostic stage; in 25 patients, BPPV was confirmed during the first visit thanks to traditionally-performed diagnostic maneuvers. In 16 patients, BPPV was ruled out through the TRV Chair, and they were subjected to subsequent diagnostic tests, while 6 people withdrew from the study. All of these individuals were excluded from the statistical calculations.

The assessment of the BPPV subtype of the patients was as follows: 7 cases of p-BPPV CAN, 8 cases of p-BBPV CUP, 6 cases of h-BPPV CAN (geotropic), 9 cases of h-BPPV CUP (apogeotropic), 5 cases of a-BPPV CAN, e cases of a-BPPV CUP, and 3 cases of multicanal changes. In 1 patient, a change in BPPV type from multicanal to h-BPPV occurred after the therapeutical maneuvers were performed. Therefore, this patient was hospitalized and treated, with positive results. In 16 patients, positional nystagmus with vertigo persisted; therefore, the therapeutic maneuvers had to be repeated. The mean number of treatment sessions required for recovery was of 2.3 ± 1.66, considering all types of BPPV ( Tables 1A B ).

**Table 1A:** Percentage of BPPV subtypes

BPPV		Number of patients	%	Total (%)
**p-BPPV**	CAN	7	17.5	37.5
	CUP	8	20	
**h-BPPV**	CAN	6	15	37.5
	CUP	9	22.5	
**a-BPPV**	CAN	5	12.5	17.5
	CUP	2	5	
**Multicanal**		3	7.5	7.5

Abbreviations: a-BPPV, anterior-canal BPPV; BPPV, benign paroxysmal positional vertigo; CAN, canalolithiasis; CUP, cupulolithiasis; p-BPPV, posterior-canal BPPV; h-BPPV, lateral-/horizontal-canal BPPV.

**Table 1B:** Percentage of BPPV subtypes

BPPV	Number of patients	%
CAN	18	45
CUP	19	47.5
Multicanal	3	7.5

Abbreviations: BPPV, benign paroxysmal positional vertigo; CAN, canalolithiasis; CUP, cupulolithiasis.

The mean DHI score before the treatment maneuvers was of 40.3 ± 20.4 points. After the maneuvers, the mean final score was of 22.2 ± 17.4 points, which constitutes an improvement of 44.91%. The difference in DHI scores was statistically significant ( *p* < 0.0001). After a thorough analysis of the questionnaire components, the most significant improvement—of 66.1%—was observed in the DHI-P ( *p* < 0.00001). The mean total score was of 17.1 ± 5.6 points before the maneuvers, whereas after the maneuvers it was of 5.8 ± 6.0 points. Another significant improvement (of 59.5%) concerned the DHI-F ( *p* < 0.0001), which changed from 17.6 ± 10.7 to 7.1 ± 7.1 points. The smallest improvement (of 34.4%) was observed regarding the DHI-E ( *p* < 0.0001), with the initial mean score of 13.5 ± 10.0 points reduced to 8.8 ± 7.7 points after the maneuvers ( Table 2 ).

**Table 2:** Mean scores on the Dizziness Handicap Inventory (DHI) and its domains

	Before treatment	After treatment	Difference (%)	*p* -value
**DHI: total**	40.3 ± 20.4	22.2 ± 17.4	44.91	< 0.0001
Functional domain	17.6 ± 10.7	7.1 ± 7.1	59.5	< 0.0001
Emotional domain	13.5 ± 10.0	8.8 ± 7.7	34.4	< 0.0001
Physical domain	17.1 ± 5.6	5.8 ± 6.0	66.1	< 0.0001

For the VSS, the mean score before treatment was of 17.7 ± 11.1 points. In the closing stage of the treatment, the score dropped to 10.4 ± 11.1 points, which corresponds to a statistically significant ( *p* < 0.0001) improvement of 41.5%. Regardinh the BBS, the initial mean score was of 51.78 ± 4.45 points, whereas, after the maneuvers, it improved by 3.4%, with a mean of 53.58 ± 4.73 points ( *p* < 0.0001) ( Table 3 ).

**Table 3:** Mean scores on the VSS and on the BBS

	Before treatment	After treatment	Difference (%)	*p* -value
**VSS**	17.7 ± 11.1	10.4 ± 11.1	41.5	< 0.0001
**BBS**	51.78 ± 4.45	53.58 ± 4.73	3.4	< 0.0001

Abbreviations: BBS, Berb Balance Scale; VSS, Vertigo Symptom Scale.

## Discussion

The current study evaluated the effectiveness of the TRV Chair in the diagnosis and subsequent treatment of difficult cases of BPPV. The results proved to be congruent with the researchers' expectations. Out of the initial group of 87 patients with undiagnosed but suspected BBPV, and after a preliminary exclusion of 25 patients by confirming BPPV during their first visit with the use of traditional diagnostic maneuvers, a correct diagnosis was made in 40 patients (45.9%). In 16 patients, BPPV was ruled out on the TRV Chair, and they were subjected to further diagnostic processes. The total number of patients excluded from the study was of 41 (47.1%), with 6 (∼ 7%) not reporting to a follow-up appointment or withdrawing from further examination.

While reviewing the available literature before commencing the study, a similar work was found relating to BPPV and the effectiveness of the TRV Chair. In a study conducted in 2020, Pedersen et al. 23 analyzed a group of 81 patients with BPPV classified as having treatment-resistant BPPV. In total, the study showed improvement in 75 (92.6%) patients. The mean number of maneuvers required to achieve improvement was of 2.23 ± 1.66. Treatment failure was observed in 6 patients (7.4%), all of them female individuals with an averageage of 78.3 years and presence of symptoms for an average of 12 months. The subtypes of BPPV included h-BPPV (66%), with an equal distribution of CAN and CUP. Additionally, 1 patient (17%) was diagnosed with p-BPPV CAN, and another patient (17%), with multicannal BPPV. In this study, 23 the patients were considered intractable because all previous treatments had been unsuccessful despite the characteristic symptomology and diagnostic test results. All patients had undergone several (at least five) individual BPPV procedures (manual repositional maneuvers, such as the standard Epley maneuver, the log rolling maneuver, and the Semont maneuver on an examination bed) before referral, and they remained symptomatic for an average of 16 months. 23 The group of patients examined in the present study had not had a diagnosis despite undergoing manually-performed diagnostic maneuvers, and the diagnosis was only facilitated later by the TRV Chair.

During the present study, the patients were observed immediately after the maneuvers, and a follow-up examination was performed 3 weeks later. In the case of persisting BPPV, the treatment in the TRV Chair was repeated, and the patient underwent another follow-up examination. This procedure was continued until clinical improvement was observed in the patient. One should bear in mind that most cases of BPPV resolve spontaneously. In the case of our group, the patients had been experiencing undiagnosable vertigo for a long time and did not experience spontaneous recovery. It is worth mentioning that the process of spontaneous recovery is often incomplete. In some patients, it may last many months and may be interspersed with periods of remission and relapses. In the elderly population, due to changes in the composition and density of the otoliths, or post-inflammatory changes in the viscosity of the gelatinous substance in which otoliths are embedded, otolith detachment occurs more frequently, which may explain the prolonged duration of BPPV and its recurring nature.

Tables 1AB show the total number of distinctive BPPV subtypes diagnosed during the study. It is worth noting that there was a relatively high percentage of patients with CUP and BPPV affecting the anterior and lateral canals, as well as a high percentage of multicanal BPPV compared to disorders occurring statistically more often in the general population. Studies 24 have reported that CAN is more frequent than CUP. Statistically, the most frequently-diagnosed form of BPPV is p-BPPV, with rates ranging from 85 to 95%. 23 25 26 In the current study, the percentage of patients diagnosed with p-BPPV was of 37.5% (15 patients), whereas CAN accounted for 46.7% (7 patients), and CUP, for 53.3% (8 patients). Studies 24 27 28 rates of CUP cases in p-BPPV ranging from 6.4 to 7.2%. Regarding lateral canal disorders, h-BPPV is statistically recognized in approximately 5 to 15% of the cases. 23 25 26 In the present sutyd, h-BPPV occurred in 37.5% (15 patients), with 6 cases (40%) of CAN and 9 cases (60%) of CUP. The pathologic condition of the anterior canal is considered to be the rarest form of BBPV, occurring only in 2% of all BBPV patients. 23 25 26 It is believed that a statistically-low percentage of this BPPV subtype may stem from diagnostic difficulties, rather than from a lower occurrence of this disorder in the general population. In the present study, a-BPPV was diagnosed in 17.5% (7 patients) of the patients, with 5 (71.4%) cases of CAN and 2 (28,6%) cases of CUP, which still indicates a rarer occurrence of this subtype of BPPV in comparison to other types. However, it is a significantly higher percentage than our team had initially anticipated. The multicanal pathologic condition affected 7.5% (3) of the examined patients, whereas in 1 patient (2.5%), a change in BPPV subtype from multicanal into h-BPPV was observed. This case should be given particular attention. Because of the performed therapeutical maneuver, the patient with multicanal BPPV developed h-BPPV with all acute symptoms, including nausea, vomiting, nystagmus, and severe feeling of vertigo, which worsened when changing body position. Therefore, we decided that the patient should be hospitalized and treated intravenously. After the symptoms subsided, therapeutic maneuvers were performed, which helped improve the patient's general condition. Our team considered this case important and worth describing in the manuscript because it illustrates that, in the case of multicanal BPPV, the treatment of patients does not always yield 100% of improvement, and in rare cases it can cause deterioration, often requiring hospitalization. The study by Baydan-Aran et al. 29 included 49 patients aged between 26 and 73 years diagnosed with multicanal BPPV. The authors analyzed and compared the effectiveness of the TRV Chair with manually-performed maneuvers. They did not observe a significant difference between the number of maneuvers performed in either group until the end of the treatment ( *p* > 0.05), with a mean of 2.57 ± 1.03 maneuvers in the treatment group, and 2.25 ± 1.16 in the TVR group. Based on the study by Baydan-Aran et al. 29 and several cases of multicanal BPPV in the present study, we can conclude that this condition is still rare and difficult to treat. In the current study, the TRV Chair was used to diagnose the previously undiagnosed condition that was the cause of vertigo in patients with negative results of diagnostic maneuvers, which also helped in the diagnosis and subsequent treatment of BPPV, including multicanal BPPV. We did not compare the effectiveness of manual maneuvers with that of the TRV Chair. In the study by Baydan-Aran et al., 29 neither the TRV Chair nor the manual maneuvers were shown as superior to one another in the treatment of multicanal BPPV cases. 29 This topic requires further research.

The results of the present study significantly differ from the occurrence of subtypes of BPPV in the general population. It needs to be noted that the current study was conducted on 40 patients and focused on a selected group of cases of vertigo which were difficult to diagnose. This may justify a relatively high percentage of less frequent subtypes of BPPV compared to the number of p-BPPV cases, and a similar proportion of CUP (47.5%) against CAN (45%). An increased number of a-BPPV and h-BPPV in comparison to the rates reported in the cross-sectional studies currently available may suggest an incomplete diagnosis during traditionally-performed diagnostic maneuvers. A thorough assessment of the sensitivity of the traditional maneuvers and of the TRV Chair to a-BPPV and h-BPPV requires further multicenter studies.

The traditional therapeutic maneuvers used in the current study were intended to achieve accurate and effective rehabilitation of patients with BPPV. Unfortunately, in the everyday medical practice, it may not be possible to perform the traditional diagnostic and therapeutic maneuvers. The difficulties may be associated with disorders of the cervical spine, excessive body weight or disabilities of the patient, or even inexperience of the clinicians. In patients with neck issues or a history of neck issues, the maneuvers can be safely performed in the TRV Chair to replace the traditional maneuvers. The patient is harmlessly supported in the TRV Chair throughout the entire maneuver, and the strain on their necks is reduced.

Moreover, the use of the TRV Chair in elderly people enables a clear assessment of whether vertigo is caused by BPPV or whether it is related to issues in the cervical spine affecting the spinocerebellar tract. To sum up, vertigo, in the case of significant disorders of the spine, remains difficult to explain due to the impossibility of precisely performing the maneuvers manually.

In heavy patients, the correct performance of the traditional maneuvers, characterized by the appropriate application of force and acceleration, can be challenging. The TRV Chair makes it possible to perform the maneuvers with constant acceleration and force parameters. It is also helpful in the diagnosis and treatment of disabled patients whose physique presents additional difficulties. During the current study, the maneuvers were performed on a patient who had been in a wheelchair since birth. Due to problems with the mobility of an adult, heavy, disabled man, other medical centers had significant problems with the correct performance of diagnostic maneuvers and, consequently, with establishing the correct diagnosis. However, it should be taken into account that the diagnosis of BPPV using the TRV Chair in patients with a negative result after traditional maneuvers does not necessarily mean an advantage of the former method over the latter. It seems perfectly reasonable, that those methods are complementary, and the TRV Chair used with the traditional maneuvers improves the detection of BPPV, opening better prospects for effective treatment.

The scales used in the present study for a comprehensive evaluation of patients with symptoms of vestibular disorders were the DHI, VSS, and BBS. The study demonstrated an improvement across all examined parameters.

The DHI helped to assess the functional impairments caused by dizziness in everyday life. The mean DHI score of 40.3 ± 20.4 obtained in the study before treatment maneuvers matches “moderate handicap.” The mean score of 22.2 ± 17.4 after the maneuvers, which constitutes a noticeable decrease of 44.91%, matches “mild handicap” and constitutes a statistically significant improvement in the patients' condition. 17 After breaking down the DHI into components, the most significant improvement concerned the DHI-P (17.1 ± 5.6 versus 5.8 ± 6.0; 66.1%), followed by the DHI-F (17.6 ± 10.7 versus 7.1 ± 7.1; 59.5%), and, lastly, the DHI-E, with a 34.4% improvement, from a mean value of 13.5 ± 10.0 to 8.8 ± 7.7 ( *p* < 0.0001). It is worth noting that, despite the clinical recovery of patients from dizziness and the fact that they noticed a diametrical improvement, the posttreatment DHI score was 0 only in 6 (15%) patients, and it ranged from 1 to 15 in 10 (25%) patients, which, according to the DHI scale, is below symptoms classified as “mild handicap.” In 60% of the patients, the results were > 15, despite a significant improvement. Like other authors, 3 30 our team attempts to justify this with the occurrence of the so-called residual dizziness (RD), defined as a sense of instability with no rotational or positional vertigo, lasting from 3 weeks to 3 months after the repositioning maneuvers. The causes of the phenomenon are believed to be central adaptation and the occurrence of otolith debris, not triggering visible nystagmus. 25 It is worth indicating that the questionnaires filled out by patients are subjective. In patients who were suffering from long-term BPPV of an undiagnosed type, the responses, despite a visible improvement, may be biased and related to their recent situation. This phenomenon may also be explained by the lowest improvement (of 34.4%) on the DHI-E. Questions related to this domain that are included in the survey (such as “Because of your problem, do you feel frustrated?”, “Because of your problem, are you afraid to leave your home without having someone accompany you?”, and “Because of your problem, have you been embarrassed in front of others?”) may evoke an association with a recent situation and keep eliciting negative emotions. It appears reasonable to believe that 3 weeks will not suffice to obtain plausible replies to all questions included in the survey, especially considering the duration of vertigo in the patients, including those with rare and difficult-to-diagnose instances of BPPV. Thus, future studies should aim to include a long-term check-up and assessment of patients.

Regarding the VSS-sf, an improvement of 41.5% was achieved. The initial mean values were of 17.7 ± 11.1, and, after the maneuvers, they were of 10.4 ± 11.1 ( *p* < 0.00001). The VSS assesses symptoms reported by patients and evaluates the frequency of symptoms of imbalance as well as the autonomic fear responses that have an enormous impact on the quality of life of the patients. 18 31 It is said that, based on the scale and the degree of intensification of the autonomic symptoms and somatic disorders, one may predict the disability grade in patients in the following months. Based on the aforementioned studies, we can state that the quality of life of the patients included in the present study has improved, not only in terms of symptoms but also in terms of anxiety. We can deduce that the quality of life of the patients significantly improved after the correctly-performed repositioning maneuvers. This topic requires further research.

In the current study, patients with BPPV were also tested with the BBS, since no similar research was found in the current scientific literature. Concededly, the BBS is useful for the assessment of imbalance in elderly patients with neurological disorders such as stroke, multiple sclerosis, traumatic brain injury, Parkinson's disease, and other conditions that may affect walking, such as lower extremity amputation. According to studies, the BBS appears to be moderately significant for the identification of imbalance in younger individuals with vestibular disorders and no history of collapsing. The scale does not imply the core cause of a problem; it may, however, help in assessing an increased risk of falls and, at the same time, facilitate the prevention of complications (such as fractures), which are particularly dangerous in elderly people. 20 22 32 The maximum score is of 56 points, which indicates functional balance. It is acknowledged that a score below 45 points indicates an increased risk of falls. In patients with a total score below 51, and with documented falls, there is a high probability of further falls. A score lower than 40 indicates an even 100% risk of a fall. 22 Given the characteristics of the disorder, the mean score of the sample of the present study before the treatment maneuvers was high (51.78 ± 4.45), which means that, at the beginning of the study, the risk of fall was low. After the maneuvers, the eman score improved marginally, but this was statistically significantly: there was an improvement of 3.4%, to the value of 53.58 ± 4.73 ( *p* < 0.0001). It is worth noting that a significant risk of falls in BPPV cases concerns the acute stage, whereas in the patients in the current the issue was a long-term one, which is why the scores were already high at the beginning of the study.

## Conclusion

The TRV Chair is a useful diagnostic and therapeutic tool for patients with BPPV, especially in cases with an unclear clinical picture of vertigo. Based on the study, we can conclude that the TRV Chair helps to detect and treat cases of BPPV that are difficult to diagnose with the use of traditional methods. It is thought that the number of patients suffering from BPPV is underestimated due to a lack of diagnosis or an incorrectly-conducted initial diagnosis. At the same time, the incidence of some BPPV subtypes also appears to be underestimated. Further research and a series of subsequent tests are required to better understand this phenomenon.
